# Embedding brain health in the curriculum: a qualitative study of primary school teacher perceptions

**DOI:** 10.1186/s12889-026-26747-0

**Published:** 2026-03-04

**Authors:** Joyce Siette, Jessica Ayyoub, Erin Mackenzie

**Affiliations:** 1 The MARCS Institute for Brain, Behaviour and Development, Western Sydney University, 160 Hawkesbury Rd, Westmead, NSW 2145 Australia; 2 School of Education, Western Sydney University, Kingswood, NSW 2747 Australia

**Keywords:** Brain health, Primary school, Teacher perspectives, Dementia risk, Public health

## Abstract

**Background:**

The growing prevalence of dementia has intensified efforts to identify modifiable risk factors that can delay its onset. Early education is increasingly recognised as a site of preventive intervention, with potential to instil health-promoting knowledge and behaviours from a young age. Despite this acknowledgement, limited research has explored how educators, central agents in curriculum delivery, perceive the integration of brain health content in schools. This study investigates primary school teachers’ perspectives on implementing brain health education in the classroom.

**Methods:**

Twenty-five teachers (*M*_*age*_ = 29.2 years, *SD* = 4.23) participated in semi-structured online interviews exploring their understandings of dementia and brain health, as well as their experiences with health-related teaching. Participants were recruited from public and independent schools across urban and regional Australian. Data were analysed using reflexive thematic analysis.

**Results:**

Three overarching themes were identified: (1) *Holistic brain health education*, reflecting teachers’ preference for holistic approaches that address physical, emotional, and cognitive wellbeing together; (2) *Pedagogical considerations*, highlighting the role of digital platforms and student-centred learning to support delivery; and (3) *Foundations for sustainable implementation*, including the need for institutional support, professional development, and parental involvement. Despite interest in integrating brain health content, participants reported barriers including a crowded curriculum, limited training, and unclear policy guidance.

**Conclusions:**

Findings reflect the untapped potential of primary schools as settings for brain health promotion. Supporting teachers through targeted training, curriculum alignment, and partnerships with health experts may assist the delivery brain health education in meaningful and sustainable ways. Our findings have relevance for broader public health discussions concerning lifelong brain health, while remaining grounded in the immediate educational context of primary schools.

**Supplementary Information:**

The online version contains supplementary material available at 10.1186/s12889-026-26747-0.

## Background

Dementia is rapidly emerging as one of the most pressing public health challenges globally. As of 2025, over 57 million people worldwide live with dementia [1]. With incidence rates rising, there is a growing imperative to develop and implement evidence-based strategies to address modifiable risk factors for cognitive decline [2]. While factors such as hypertension, smoking, and physical inactivity are well established, educational attainment has also been identified as a significant early determinant of long-term cognitive health [3]. Addressing educational disparities and embedding brain health literacy from an early age may offer a promising pathway toward dementia prevention on a population level [4].

Brain health literacy, defined as the ability to access, interpret, and apply knowledge about brain function and its relationship to overall wellbeing, represents a relevant construct in preventative health [5]. Individuals with higher levels of brain health literacy are more likely to adopt behaviours that support cognitive function across the lifespan [6]. As such, embedding brain health education into school curricula provides a potentially powerful means of equipping children with the foundational knowledge and habits that promote lifelong brain health. Early interventions in this domain have the potential to reduce risk factors associated with dementia, such as physical inactivity, poor nutrition, and reduced social engagement [7, 8].

Alongside the benefits in reducing the risk of dementia, the delivery of brain health education in schools can also improve a child’s overall neurodevelopment. Early interventions targeting cognitive, emotional, and lifestyle factors have the potential to enhance executive functioning, memory, attention, and self-regulation skills of children [9–11], which has long been recognised as supportive of learning and academic success [12–18]. By framing brain health promotion beyond the prevention of dementia, educators can emphasise more holistic neurodevelopment and build lifelong habits that support physical, cognitive, and emotional wellbeing. Indeed, within the Australian context, nutrition, physical activity, sleep hygiene, and emotional regulation is regularly discussed in the national curriculum in order to provide children with practical skills and knowledge that enhance overall brain function and development [19].

Adopting a developmentally-informed approach further aligns educational strategies with a child’s cognitive and socio-emotional capacities. This broader perspective encourages a proactive stance on mental health, resilience, and adaptive coping strategies and can equip children with the appropriate tools to navigate challenges across their lifespan [5, 20, 21]. Therefore, brain health education in early elementary school represents not only a preventative strategy against future cognitive decline but also a foundational component of optimal neurodevelopment and lifelong learning [22].

In childhood, education functions not only as a vehicle for cognitive and physical development but also as a foundation for enduring health practices. Evidence suggests that the impact of educational interventions is most pronounced when implemented during the formative years, particularly during pre-kindergarten and primary school [20, 21, 23]. Multi-component programs that integrate physical activity, social-emotional learning and nutrition have demonstrated positive effects on cognitive resilience, emotional well-being and academic performance [24–26]. These programs signal an opportunity to extend their scope to include brain health literacy and to embed a preventative health lens into educational policy and practice.

However, the effective implementation of brain health education in schools is not without challenges. Teachers, as primary facilitators of children’s learning experiences, are essential to the success of such initiatives. Research suggests that while educators recognise the value of health education, common barriers include a lack of resources, limited training, and insufficient institutional support [27]. In parallel, parental involvement in supporting brain health messages at home remains inconsistent. Many parents report limited confidence in their own understanding of brain health, which may constrain their capacity to reinforce school-based learning [20]. Addressing these gaps will require coordinated, inclusive strategies that support both educators and families.

The current literature on brain health education is predominantly quantitative, often focused on program outcomes rather than the lived experiences of those who deliver them. While several studies have highlighted the benefits of early childhood education programs in promoting cognitive and emotional wellbeing, few have examined teachers’ perspectives on the feasibility and relevance of such programs in everyday classroom settings [28]. In response to this gap, the current study aims to explore primary school teachers’ perspectives on integrating brain health literacy into the curriculum. Specifically, the research will investigate enablers and barriers teachers encounter, their perceived responsibilities, and their views on the resources needed to deliver such content effectively. This study is further informed by the Theory of Planned Behaviour (TPB), which proposes that individuals’ intentions to perform a behaviour are influenced by their attitudes toward the behaviour, perceived behavioural control, and subjective norms [29]. This theory has been widely applied in health and educational research to understand how individual beliefs interact with structural and contextual factors to shape practice. In the context of primary education, TPB provides a useful framework for examining how teachers’ perceptions, institutional expectations, and perceived constraints influence the implementation of health-related educational initiatives, including those related to brain health. By centring teachers’ voices, this research thus aims to inform the design of practical, scalable strategies to embed brain health education into school settings.

## Methods

### Participants

Participants were recruited by word-of-mouth, social media advertisements in Facebook teaching community groups, and individual email contact with private school administrators across Australia. To be eligible for the study, participants had to be aged over 18 years, currently employed as a primary school teacher in Australia (either part-time or full-time), able to communicate in English, and willing to provide informed, written consent. The study received ethics approval from the Western Sydney University Human Research Ethics Committee (reference H15440).

## Materials

### Demographics survey

The demographic survey included fourteen questions about the participants’ age, marital status, educational background, years of teaching experience, and information about their current teaching circumstances including role, school size, grades taught, and type of school (e.g., public, private).

### Semi-structured interview guide

The interview guide consisted of twenty open and close ended questions exploring teachers’ perspectives on brain health education for primary school-aged students (see Supplementary Material A). The questions were informed by the literature on healthy behaviours, existing brain health programs, teachers’ roles in influencing students’ lifestyles, and strategies for integrating such programs into school settings [3, 6]. Additionally, the questions expanded on previous literature exploring potential barriers and motivators that impact the implementation of positive lifestyle habits [21]. The interview questions were pilot tested on several members of the research team (including educators) to assess their validity, feasibility, and structure, and were refined following feedback. Although the interview guide introduced the topic through reference to dementia, it is important to clarify that dementia and brain health are related but distinct constructs. For this study, we refer to dementia as an umbrella group of clinical syndromes characterised by progressive cognitive decline that interferes with daily functioning, most commonly occurring in later life. In contrast, brain health is a broader, lifespan-oriented concept encompassing cognitive, emotional, and neurological wellbeing, and includes factors that support optimal brain development and functioning across childhood, adulthood, and older age [5].

The inclusion of dementia in the opening interview questions reflected the wider public health discourse linking modifiable lifestyle behaviours across the life course to reduced dementia risk. However, the interviews were not confined to dementia prevention or risk reduction. As discussions progressed, participants were asked to engage more broadly with the concept of “brain health” and was often reframed in developmentally appropriate ways relevant to primary-aged children. Teachers were encouraged to speak about nutrition, sleep, physical activity, emotional regulation, learning strategies, and overall wellbeing as components of supporting children’s brain development (see Supplementary Material). Thus, while dementia prevention discourse informed the initial framing of the interview guide, the study sought to explore teachers’ understandings of brain health in a broader educational and developmental context.

### Procedure

Participants expressed interest by contacting the research team via email after viewing the study flyer. They were then scheduled for a Teams interview and provided with an information sheet, interview questions, and a consent form to return before the meeting. At the interview’s start, participants were briefed on the process and gave verbal consent to record for transcription. Demographic questions preceded the interview. Upon completion, participants received a $20 Prezzee eGift card. Interviews lasted between 20 and 45 min.

### Data analysis

All interviews were audio-recorded and transcribed verbatim via the transcription software Otter.ai. Transcripts were reviewed for accuracy, where identifiable information was removed to maintain participant confidentiality. Data saturation was reached through an iterative process consistent with established qualitative research standards. Recruitment initially followed a purposive sampling strategy to ensure that participants represented the full range of perspectives relevant to the study phenomenon. All interviews were conducted by a single researcher (JA) trained in qualitative interviewing techniques. The interviewer had a background in educational psychology, which informed their understanding of the school context while maintaining a reflexive approach throughout data collection. Using one interviewer supported consistency in interview delivery, questioning style, and rapport across participants. As interviews progressed, the research team engaged in concurrent data collection and analysis to allow for emerging insights to guide subsequent sampling decisions. By the 20th interview, our team noted substantial repetition in participants’ accounts, with no new conceptual categories appearing during open coding. The final five interviews (participants 21–25) were conducted intentionally to test the robustness of this preliminary saturation. During these additional interviews, the data continued to confirm previously identified themes without introducing novel codes, variations, or contradictions. After the 25th participant, the team concluded that thematic categories were sufficiently rich, well-developed, and consistent across multiple cases and thus met the criteria for code saturation (i.e., no new codes) and meaning saturation (i.e., no new insights regarding the dimensions or properties of existing themes). Because additional interviews were unlikely to meaningfully extend the analytic framework, data collection was closed.

Data was analysed using reflexive thematic analysis, following Braun and Clarke’s [30] six-phase methodological framework, and managed with NVivo (Version 14). Two independent researchers (JS, JA) initially familiarised themselves with the data by repeatedly reading the transcripts to gain a deep understanding of the content. During this phase, initial thoughts were noted to inform subsequent coding. The researchers then systematically generated data-driven codes aligned with the study’s aims. These codes captured both barriers (e.g., “We are very time poor”) and facilitators (e.g., “Parental involvement helps”) relevant to the implementation of future brain health educational programs. Following initial coding, related codes were clustered into potential themes and subthemes. Themes were then reviewed iteratively to ensure internal coherence, consistency across the dataset, and conceptual clarity. For instance, codes pertaining to digital tools and cooperative efforts among stakeholders were consolidated into broader thematic categories such as “Leveraging technology and collaboration.” The emerging thematic structure was then refined and formally defined, with attention paid to semantic and latent meanings within the data. The final thematic map was reviewed by a senior qualitative researcher (EM), who confirmed the credibility and coherence of the coding. No discrepancies between coders were identified, thereby supporting intercoder reliability. The final themes and subthemes were synthesised narratively. While the analysis was primarily reflexive, the interpretation of themes was informed by the COM-B framework [31] to support theoretical sensitivity when examining teachers’ attitudes, perceived constraints, and contextual influences on implementation.

## Results

### Participant demographics

A total of 25 participants were included in this study, with a mean age of 29.2 years (SD = 4.23; range = 24–47). The majority identified as female (*n* = 16; 65%), slightly more than half were married (52%), and the majority (60%) had children, with most having either one (36%) or two children (16%). The average age of participants’ children was 6 years (SD = 3.61; range = 2–17).

In relation to educational attainment, a substantial proportion of participants held a master’s degree (72%), while the remaining 28% held a bachelor’s degree. Teaching experience ranged from 2 to 10 years (M = 5.71, SD = 2.35), with the largest proportion of participants reporting either four (20%) or six (20%) years of teaching experience. Nearly all participants (92%) were currently working as classroom teachers, with one participant each holding an administrative or curriculum release role.

Participants were employed across a variety of school sizes, with the most common being schools with enrolments of 500–599 students (32%), followed by 300–399 (20%) and 700–799 (16%). The number of students taught varied considerably, ranging from 10 to 50 students (M = 22.52, SD = 7.88), with the most frequently reported student numbers falling between 20 and 24 students (32%).

In terms of grade level, most participants were teaching Stage 1 (Years 1–2; 40%) or Stage 2 (Years 3–4; 44%), while a smaller proportion taught Stage 3 (Years 5–6; 12%) or across all primary stages (K-6; 4%). All participants were teaching at a single school site. The majority were employed in public schools (60%), followed by private (32%) and Catholic schools (8%). Table 1 shows the demographic information for all participants in this study.

**Table 1:** Demographic characteristics of participants (*N* = 25)

*Variable*	*N*	*%*	*M (SD), Range*
Age			29.2 (4.23), 24–47
Gender			
Female	16	65%	
Male	9	36%	
Marital status			
Married	13	52%	
Single	11	44%	
De facto	1	4%	
Number of children in the family			
0	10	40%	
1	9	36%	
2	4	16%	
3	1	4%	
4	1	4%	
Average age of children in the family			6 (3.61), 2–17
Highest education level			
Bachelors	7	28%	
Masters	18	72%	
Years spent teaching			5.71 (2.35), 2–10
2	3	12%	
4	5	20%	
5	4	16%	
6	5	20%	
7	2	8%	
8	1	4%	
9	1	4%	
10	3	12%	
Current role or position			
Classroom teacher	23	2%	
Administrator	1	4%	
Curriculum release	1	4%	
Size of the school			
300–399	5	20%	
400–499	1	4%	
500–599	8	32%	
600–699	1	4%	
700–799	4	16%	
900–999	1	4%	
1000–1499	3	12%	
1500–2000	2	8%	
Number of students taught			22.52 (7.88), 10–50
10–14	3	12%	
15–19	6	24%	
20–24	8	32%	
25–29	5	20%	
30–34	2	8%	
50	1	4%	
Grade/s currently teaching			
Stage 1 (Years 1–2)	10	40%	
Stage 2 (Years 3–4)	11	44%	
Stage 3 (Years 5–6)	3	12%	
All stages (K-6)	1	4%	
Number of schools teaching at			
One	25	100%	
Type of school			
Private	8	32%	
Catholic	2	8%	
Public	15	60%	

### Main themes and subthemes

Three overarching themes emerged, with each comprising several interconnected subthemes. These themes reflect the multidimensional perspectives of educators on brain health promotion in primary schools. A total of eight subthemes were identified across the three main thematic categories. Table 2 presents a summary of the primary themes and their associated subthemes.

**Table 2:** Summary of main themes and subthemes

Main Themes	Subthemes	Mapped COM-B Elements
Holistic brain health education	Physical and cognitive engagement	Skills (practical application); Memory/attention/decision processes (reducing cognitive load); Behaviour regulation (goal-setting, planning)
	Nutrition and sleep	Knowledge (lifestyle factors); Behaviour regulation (self-monitoring, routine maintenance); Psychological capability (cues, simplified information)
	Emotional regulation and wellbeing	Emotion (fear, anxiety, enjoyment); Beliefs about capabilities (self-efficacy); Beliefs about consequences (impact of stress/emotions); Optimism (positive affect enabling change)
Pedagogical considerations	Technology as a double-edged sword	Environmental context/resources (tools that help or hinder), Memory/attention processes (need for clarity and cognitive support)
	Student-centred learning	Skills (balancing structure/autonomy); Intention (proactive learning); Social/professional identity (individual-aligned learning); Knowledge (reinforcement and extension)
Foundations for sustainable program implementation	Time and financial constraints	Physical opportunity (time, resources); Behaviour regulation (tools for managing competing demands)
	Teacher training and development	Reinforcement (feedback); Skills (teaching and scaffolding behaviour change); Beliefs about capabilities (educator confidence)
	Parental involvement	Social opportunity (family networks); Social influences (modelling, encouragement); Emotion and reinforcement (accountability and motivational support)

#### Theme 1: holistic understandings of brain health

The first overarching theme that emerged from the data was the conceptualisation of brain health as a holistic and multidimensional construct. Teachers consistently articulated an understanding of brain health that transcended narrow cognitive definitions, instead encompassing the interrelated domains of physical activity, cognitive engagement, nutrition, sleep, and emotional regulation. This integrative perspective reflects a systems-level understanding, wherein various aspects of a child’s physical and psychosocial environment were seen to coalesce in support of optimal neurodevelopment and wellbeing.

Teachers did not perceive brain health as an isolated domain to be taught in silos, but rather as an intrinsic component of daily pedagogical practice. One participant succinctly stated, “It’s not just a formal program; we weave it into everyday activities,” to highlight the implicit integration of brain health concepts across routine school interactions. The three subthemes below outline how participants conceptualised and enacted brain health through *physical and cognitive engagement*, *nutrition and sleep*, and *emotional regulation and wellbeing*.

### Physical and cognitive engagement

Participants widely recognised the symbiotic relationship between physical movement and cognitive functioning, and consistently noted that brain health cannot be nurtured without simultaneous attention to both. One teacher remarked, “Physical activities and playing games that stimulate the brain are the most effective strategies for encouraging healthy behaviours,” which reflected the prevailing belief that embodied learning practices enhance attentional focus and executive function. Teachers also described a bi-directional relationship between physical health and mental alertness, “Physical health is connected to mental alertness,” affirming their belief that movement primes the brain for learning.

This subtheme also reflected teachers’ observed improvements in classroom behaviour and academic engagement when physical activity was integrated into the school day. One participant shared, “I see a positive response from students when they engage in physical activity before class,” demonstrating an awareness of how kinesthetic engagement can serve as a regulatory strategy that promotes readiness to learn. Several teachers highlighted the value of activities that combine physical exertion with cognitive demand, such as games that require memory, sequencing, or problem-solving, and described these practices as suitable in promoting holistic brain health.

### Nutrition and sleep

Teachers identified nutrition and sleep as essential physiological substrates of cognitive function and mental wellbeing. Participants emphasised the need to embed education around healthy eating and adequate rest into the broader school curriculum and recognised that these elements were foundational to students’ capacity for learning and emotional regulation. One teacher explained, “By making students know the kind of food they should eat, the importance of exercise and having adequate sleep… the brain will be able to function.” This captured the consensus that these behaviours were not ancillary but central to academic and developmental success.

Despite recognising their importance, several participants reported persistent challenges in addressing entrenched unhealthy dietary behaviours, particularly those rooted in familial or cultural norms. One teacher shared, “My major struggle is with kids that have been struggling with unhealthy eating habits… they’ve been indulged for very long.” In response, teachers adopted proactive measures such as allocating time during lunch to ensure students consumed their meals and had a demonstrable commitment to modifying the school environment in support of better nutrition practices.

### Emotional regulation and wellbeing

Emotional wellbeing and regulation were perceived not only as components of brain health but also as key life skills necessary for social functioning and academic success. Teachers consistently advocated for the intentional teaching of emotional literacy and regulation strategies, including structured classroom interventions and informal opportunities for emotional expression. One teacher encapsulated this by stating, “Life is about how they respond to situations, not just the experiences,” and emphasised the importance of resilience and emotional adaptability.

A range of practices were employed to support emotional development. This ranged from the establishment of “calm down corners” to direct instruction on the neurobiology of emotions and regulation techniques. One participant reported teaching students about “different parts of the brain… and strategies to manage emotions like breathing exercises,” which reflected a growing pedagogical shift toward neuroeducation. Importantly, teachers also challenged prevailing cultural stigmas around emotional expression. As one teacher shared, “Punching pillows, writing things down… it’s actually okay,” where the act of normalising emotional expression was framed as central to mental wellbeing and as a means of “rewiring the narrative” surrounding emotional control in young learners.

#### Theme 2: pedagogical considerations

A second overarching theme centred on the pedagogical mechanisms through which brain health programs could be effectively implemented and sustained in primary school settings. Teachers identified both facilitators and constraints to implementation, with particular emphasis on the roles of technology and student-centred pedagogies. Two subthemes emerged within this domain: *Technology as a double-edged sword* and *student-centred learning.*

### Technology as a double-edged sword

Teachers described technology as a tool with significant pedagogical potential for enhancing engagement with brain health content. The use of interactive digital platforms, artificial intelligence, and multimedia resources was seen to demystify complex topics and make health education more accessible. One teacher noted, “Technology can be leveraged through digital learning tools, interactive simulations, and online resources that make brain health engaging and accessible,” while another emphasised its motivational value: “Exciting pictures and diagrams are very attention-catching for kids.” The integration of artificial intelligence (AI) was also viewed as a promising development, with one participant asserting that “With the use of AI, brain health can be enhanced, promoting social and emotional wellbeing”.

However, this enthusiasm was tempered by concerns regarding overuse. Participants expressed caution about the detrimental effects of excessive screen time and the displacement of physical activity. As one teacher put it, “Technology needs to be balanced with other activities … I encourage them to limit their screen time and focus more on physical activities and reading”, which indicated a need for guidance to manage digital exposure so brain health interventions remain effective.

### Student-centred learning

The second pedagogical subtheme focused on adapting the learning environment to meet the individual needs of students to ensure they are active participants in their own learning process. Teachers highlighted the necessity of tailoring programs to suit the unique characteristics of each student, recognising that a one-size-fits-all approach may not be effective and that a personalised learning experience is more suitable:

Every child is different and there are certain things that are unique to a child… what I basically do is try to work with a child according to the features that are unique to that child.

While most participants supported involving students in the co-design of educational experiences, opinions varied on the extent of this involvement. Some teachers championed student agency and viewed it as central to engagement and self-esteem, while others expressed reservations about students’ decision-making capacity, preferring to defer to parental or professional judgment. A balanced perspective was offered by those who encouraged joint decision-making processes involving both students and caregivers. As one teacher stated, “Yes, absolutely, they should be involved because they are the ones who are learning, and they have the right to know and understand”. However, some teachers cited concerns about their developmental stage and ability to make informed choices for their educational needs, “I don’t involve students in decision-making because they might not have the best ideas for themselves. Teachers and parents know their strengths and weaknesses”.

Teachers also highlighted the importance of inclusive design for students with disabilities, and advocated for one-on-one support, trained personnel, and curriculum modification to meet diverse learning needs: “I ensure programs for students with disabilities are adapted through one-on-one interaction, employing trained personnel, and tailoring the curriculum to meet their needs”.

#### Theme 3: foundations for sustainable program implementation

The final theme pertained to structural enablers and barriers to the sustainable implementation of brain health programs in primary schools. Teachers pointed to critical needs in three subthemes: *time and financial constraints, teacher training and development,* and *parental involvement.*

### Time and financial constraints

One of the most prominent challenges teachers described facing in implementing future brain health programs was the constraint of time and financial resources. Teachers frequently expressed frustration at the lack of time within the school day to incorporate health education initiatives, often due to the prioritisation of core curriculum subjects like Mathematics and English. As one teacher explained, “We are very time poor. We’re under a lot of pressure. We’re constantly testing then giving feedback. Maths and English are priorities, and we have to fit so many extra things”. Another added, “Time… sometimes other curriculum subjects take over priority”, which further highlights the difficulties in balancing brain health programs with existing academic demands.

Financial limitations were also raised as a core barrier to effective program development and implementation. Teachers highlighted the importance of adequate funding, describing that such programs require both materials and staffing support, neither of which can be sourced without proper financial backing. One teacher commented, “Funding is very important… because you know, as a teacher, you can’t do that out of your pocket”. Participants often explained how financial constraints led to insufficient staffing and training which will further impede their ability to implement and sustain brain health programs effectively. Another teacher discussed the wider impact of financial budget cuts explaining:

Cost is a big one at the moment because budgeting is really tight and that puts strain on the school and the principal to make cuts in areas that they think are not as important, whereas others might think that they are very important… not just the training of staff, but the lack of staff.

### Teacher training and development

This subtheme reflected the necessity of teacher training and continuous professional development opportunities to ensure effective integration of brain health into the curriculum. Teachers expressed concern over the lack of structured and ongoing training opportunities, particularly for new staff. There was a general reflection of insufficient follow-up training, which can lead to inconsistent implementation and reduced program impact over time. As one teacher shared:

Like we did this rock and water training back in term three last year, and it’s never been mentioned again. So, when new staff come in, they wouldn’t have ever heard of it, so I think that’s definitely a challenge.

Teachers consistently advocated for more comprehensive training that incorporates the latest neuroscience, pedagogy, and practical strategies for integrating brain health into the curriculum. One teacher expressed, “We need training, but we also need more staff to help run these programs”, while another stressed the need for training in specific areas, “Training of staff is important as well as having enough time and resources to implement programs effectively”. Teachers also highlighted the need for educational resources that cover a range of concepts including child development and teaching methods, to ensure effective integration of brain health programs. As one participant suggested, “Professional development should cover the latest brain science, effective teaching methods, and strategies for integrating brain health into the curriculum”. Another teacher added, “Attending seminars on brain health, earning certifications in child psychology, and participating in online sources can help teachers stay informed”. These insights reflect the importance of equipping teachers with the knowledge and tools they need to deliver brain health programs effectively and sustainably.

### Parental involvement

Parental engagement was consistently framed as a linchpin of effective and sustained brain health promotion. Rather than viewing parents as peripheral, educators framed them as co-facilitators of health promotion, with responsibilities extending beyond school grounds. Strategies such as assigning take-home activities that actively involved parents were seen as effective in encouraging reinforcement of brain health concepts at home. As one teacher noted, “You engage the parent by giving homework that involves them… a parent could be obliged to support it”. This shared responsibility was viewed not only as a means of reinforcing behavioural messages but also as a mechanism to foster deeper, more consistent understanding of brain health across contexts.

Open, reciprocal communication between teachers and families further strengthened these efforts. Participants described a range of strategies (e.g., parent-teacher meetings, newsletters, one-on-one discussions) to maintain parental awareness and engagement. This transparency permitted for early intervention when concerns about student wellbeing arose and encouraged co-learning, with some educators reporting that they actively teach parents during school events to build shared knowledge. As one participant put it, “We highlight these issues during parent-teacher association meetings, teaching the parents as well so they can guide their children”. Importantly, these forms of parental involvement were often embedded within a broader call for collaborative, community-oriented approaches. Teachers discussed the importance of alignment among educators, families, and external stakeholders and often framed brain health promotion as a collective responsibility. The development of a shared vision was seen as essential: “Developing a sense of community between parents, teachers and community members is key… they should have a shared vision”. Digital tools such as community apps and online surveys were also proposed as promising strategies to facilitate two-way communication and promote shared ownership.

Teachers also emphasised the need for culturally sensitive and trust-building approaches, and particularly so when working with families from diverse backgrounds. Taken together, these insights position family-school partnerships not only as facilitators of program implementation but as central components of inclusive, community-led brain health promotion.

## Discussion

The integration of brain health education into primary school settings presents an opportunity to address the holistic development of children during a critical window of neurodevelopment. This study explored primary school teachers’ attitudes towards brain health educational programs, their perceived facilitators and barriers to implementation, and the broader implications for educational policy and practice. Findings showed strong support for holistic, integrative approaches that encompass cognitive, emotional and physical wellbeing. However, implementation was perceived to be constrained by limited time, insufficient training and scarce educational resources. These findings highlight both teachers’ motivation to engage with brain health education and the contextual factors that shape their capacity to do so.

However, it is important to clarify the conceptual distinction between dementia and brain health within the primary school context. Although dementia informed the initial framing of the interview guide, it represents a specific clinical syndrome characterised by progressive cognitive decline, most commonly occurring in later life. In contrast, brain health is a broader, lifespan-oriented construct encompassing the cognitive, emotional, and neurological processes that underpin functioning and wellbeing from early childhood onwards [5]. While modifiable risk factors across the life course are increasingly recognised within dementia risk reduction discourse [3], brain health education in primary schools should not be conceptualised as early dementia risk reduction in a narrow clinical sense. Our findings indicate that teachers did not interpret brain health education as teaching children about dementia. Rather, they framed brain health in developmentally appropriate terms and emphasised sleep, nutrition, physical activity, emotional regulation, attention, and learning readiness. From a public health perspective, this distinction is acute. Positioning brain health as part of holistic child development aligns with existing whole-school wellbeing frameworks and avoids medicalising primary education [32]. Instead, it situates brain health within universal health promotion strategies that support optimal neurodevelopment, educational attainment, and long-term population health outcomes.

Teachers’ positive disposition toward brain health education aligns with Ajzen’s Theory of Planned Behaviour [29], which posits that favourable attitudes toward a behaviour influence the intention to act. In this study, participants expressed positive intentions towards brain health initiatives and viewed them as valuable, relevant, and compatible with existing pedagogical practices. Rather than perceiving brain health education as an additional burden, teachers commonly described its practice which could be embedded within routine classroom activities. This preference reflects constructivist theories of education, which hold that learning is most effective when situated in authentic, lived student experiences [33, 34]. Strategies such as movement breaks, mindfulness routines, and peer-led emotional discussions were commonly cited as both feasible and effectively integrated within current pedagogical practices. This integration also reframes wellbeing as core to educational outcomes, not peripheral. Rather than treating mental and emotional health as secondary concerns, teachers advocated for their inclusion in daily pedagogy. This reflects growing expectations that schools design integrated and comprehensive approaches to supporting student health and wellbeing (e.g. The Wellbeing Framework for Schools [35]), whilst at the same time recognising the critical interrelationship between health, wellbeing, and learning.

Despite their willingness, teachers articulated several challenges. Chief among these was the lack of available time within an already crowded curriculum. Participants described how the pressure to meet academic benchmarks, particularly in preparation for standardised assessments, often reduced the space available for more holistic or preventive educational content. This dilemma is echoed in wider literature, which notes that even when teachers value health promotion, competing instructional demands can crowd out such efforts [27]. While enthusiastic, many educators also felt underprepared to teach brain health concepts in age-appropriate and pedagogically sound ways. Without access to professional development or structured guidance, implementation often relied on individual trial and error and this can risk inconsistency and diminished confidence.

Technology emerged as both a facilitator and a concern. Teachers saw digital tools as opportunities to engage students through interactive learning, in line with research suggesting that gamified platforms can boost motivation and achievement [36, 37]. Yet, they also warned against overreliance on screens, citing growing concerns over excessive screen time and its adverse effects on academic performance, sleep, and emotional wellbeing [38]. These mixed views reflect the importance of balanced digital integration in educational program design.

Teachers also expressed facing challenges in reversing unhealthy lifestyle habits formed in early childhood. This concern aligns with existing research in developmental psychology, which highlights those habits formed in early years tend to be more resistant to change due to their deep integration into a child’s daily routine and identity [39]. Teachers’ observations reflect this challenge, as they often encountered students whose dietary habits were influenced by their family environment, cultural norms and socioeconomic status. However, reversing these entrenched behaviours requires not only school-based interventions but also collaboration with parents and the broader community. Indeed, the most effective interventions in modifying children’s dietary habits often involve whole-family approaches rather than focusing solely on the individual child [40]. This tension between the desire to promote healthier lifestyle choices and the practical limitations teachers face suggest the importance of leveraging other tools and parental involvement to enhance the delivery of brain health programs.

### Implications

Our findings highlight the importance of systematic changes including greater financial investment, professional development, stakeholder collaboration and innovative approaches for the successful and sustainable implementation of educational programs in primary schools. Teachers are encouraged to embed brain health practices into daily classroom activities, such as incorporating regular brain breaks comprising of physical movement and emotional regulation techniques throughout the school day [41]. While time constraints emerged as a significant perceived barrier to implementation, integration of these short, practical routines (rather than longer, stand-alone brain training programs) may represent a realistic and practical option for scalable brain health promotion in schools. The findings suggest that increased funding and resource allocation may support teachers’ capacity to implement brain health initiatives without being constrained by systemic limitations. Rather than positioning responsibility solely at the individual teacher level, the findings suggest that institutional support, such as guidance materials, training opportunities, and shared framework, may enhance perceived capacity to implement brain health initiatives effectively. Thus, collaboration between training institutions and education departments to design professional development courses on brain health practices are also encouraged, as is pre-service teacher education in this area. Networks that encourage consistent communication between parents and teachers should be prioritised to ensure brain health practices are reinforced at home. At the school system level, communication with parents about brain health may facilitate widespread and consistent messaging without additional imposts at the individual teacher and school level. Finally, ongoing feedback from students, parents, and other stakeholders is likely to enable consistent and sustainable development of brain health programs.

### Strengths and limitations

The present study includes several strengths and weaknesses that must be considered. A strength is the considerable sample size within a qualitative methodology, where the use of semi-structured interviews supported a more in-depth exploration of teachers’ unique experiences and perspectives towards brain health program implementation. Furthermore, the participant pool included individuals with diverse professional backgrounds and varying years of experience, representing a balanced mix of private, Catholic, and public school teaching, provided a wider range of perspectives and experiences. However, perspectives from other stakeholders, such as school policymakers, or parents, were lacking. Future research should adopt a multi-stakeholder approach to understand broader systemic factors and assess the long-term impact of brain health education on student outcomes. Furthermore, while the study explored teachers’ perspectives on implementing brain health education, information regarding their prior experience or specific training in this topic was not collected. Consequently, we are unable to determine how differences in teaching background or formal training may have influenced participants’ views or approaches. Future studies should consider collecting detailed information on participants’ professional experience and relevant training to better contextualise findings.

## Conclusion

Despite brain health programs’ alignment with progressive educational ideals, our study identified several structural challenges and institutional barriers that hinder the effective implementation of such initiatives. Time limitations, financial constraints, and a lack of sufficient training and professional development were repeatedly identified as significant obstacles. Together, our findings highlight the necessity for a coordinated, multi-level strategy that addresses both the material and ideological conditions shaping teachers’ work. Future research should continue to explore the specific tools, resources, and institutional supports required to fully actualise the potential of brain health education. Such exploration is essential not only for enhancing the physical, emotional, and cognitive capacities of students but also for addressing broader systematic and institutional factors that shape how educational programs are prioritised and implemented within school contexts.

## Supplementary Information

Supplementary Material 1.

## Data Availability

The data underlying this article will be shared on reasonable request to the corresponding author. The data are not publicly available due to ethical and privacy restrictions.
