# Supplementary material for: Embedding brain health in the curriculum: a qualitative study of primary school teacher perceptions
Source: BMC Public Health. 2026 Mar 4;26:1182. doi: 10.1186/s12889-026-26747-0 (PMC13067744; doi:10.1186/s12889-026-26747-0)
Supplement: Supplementary file 1 — Supplementary Material 1. [file 12889_2026_26747_MOESM1_ESM.docx]

**Supplementary Materials**

*Supplementary Material A. Semi-structured interview guide.*

| **Section** | **Question** |
| --- | --- |
| Introduction | 1. Can you share what you know about dementia? 2. Can you share what you know about brain health? 3. Can you share your experience with implementing health and wellness programs in primary school settings? |
| Teaching strategies | 1. How do you currently promote brain health and healthy lifestyles among your students? 2. How would you explain brain health to a primary school-aged child? Would this definition differ between stages? Would you explain this differently to a child who is neurodiverse?  *Moderators: If yes, why and how?* 3. What are the key challenges you face in implementing health education programs in schools? 4. How do you engage parents in supporting brain health initiatives at home? 5. Have you noticed any differences in the acceptance of brain health (or general health) programs among different cultural or socio-economic groups? 6. What strategies do you find most effective in encouraging students to adopt healthy behaviours?  *Moderators: Probe for:*    1. *Physical exercise*    2. *Eating well*    3. *Socialising*    4. *Looking after their mental health*    5. *Sleeping well*    6. *Being cognitively active/challenged* 7. Can you share any success stories or positive outcomes from previous health promotion initiatives? |
| Pedagogical considerations | 1. How do you think technology can be leveraged to enhance brain health education in schools? 2. In your opinion, what are the most important factors to consider when designing brain health programs for primary school-aged children? 3. How do you assess the effectiveness of health and wellness programs in your school? 4. What resources or support do you feel are necessary to effectively implement brain health programs in primary schools? *Moderators: Probe for:*    1. *Cost/expenses*    2. *Training of staff*    3. *Who will deliver it*    4. *Lack of experience*    5. *Lack of space for activities*    6. *Time* |
| Recommendations | 1. What strategies would you use to encourage collaboration and communication among school staff, parents, and community stakeholders if we were promoting brain health?    1. Would you involve students? How do you involve students in decision-making processes related to health education programs?    2. How do you handle resistance or scepticism from staff or parents regarding health education programs? 2. How do you ensure that future brain health education is integrated into the curriculum effectively? 3. What strategies can you suggest to make future brain health education engaging and interactive for students? 4. What ongoing professional development opportunities do you think are important for educators in promoting brain health? 5. How do you measure the impact of health education programs on student outcomes and behaviour change? 6. How would you adapt health education programs to meet the diverse needs of students with disabilities or special needs? |

*Supplementary Material B. Codebook mapping initial codes to themes, sources, and references.*

| **Name** | **Themes** | **Sources** | **References** |
| --- | --- | --- | --- |
| acceptance of programs | Theme 3c | 1 | 1 |
| accessibility | Theme 2b | 1 | 1 |
| adaptability | Theme 2b | 1 | 1 |
| adapting for neurodivergent students | Theme 2b | 1 | 1 |
| awareness | Theme 2 | 1 | 1 |
| behaviour | Theme 1c | 1 | 1 |
| behavioural challenges | Theme 1c, 2b | 1 | 1 |
| behavioural changes | Theme 1c | 1 | 1 |
| brain functionality | Theme 1 | 1 | 1 |
| brain health education | Theme 1 | 1 | 1 |
| brain health engagement | Theme 1, 2b | 1 | 1 |
| brain health importance | Theme 1 | 1 | 1 |
| brain health knowledge | Theme 1 | 1 | 1 |
| brain health promotion | Theme 1 | 2 | 2 |
| challenges in implementation | Theme 3a, 3b | 3 | 3 |
| challenges in understanding | Theme 2b, 3c | 1 | 1 |
| child-centred | Theme 2b | 1 | 1 |
| cognitive challenges | Theme 1a | 1 | 1 |
| cognitive growth | Theme 1a | 1 | 1 |
| collaboration | Theme 2a, 3c | 3 | 3 |
| communication | Theme 3c | 6 | 6 |
| community | Theme 3c | 1 | 1 |
| community involvement | Theme 3c | 1 | 1 |
| community support | Theme 3c | 1 | 1 |
| comprehensive approach | Theme 1 | 1 | 1 |
| continuous training | Theme 3b | 1 | 1 |
| cost | Theme 3a, 3b | 2 | 2 |
| cost to parents | Theme 3a, 3c | 1 | 1 |
| cultural acceptance | Theme 2b | 1 | 1 |
| cultural differences | Theme 2b, 3c | 5 | 5 |
| cultural sensitivity | Theme 2b | 1 | 1 |
| curriculum priorities | Theme 3a | 1 | 1 |
| decision-making | Theme 2b | 1 | 1 |
| digital learning tools | Theme 2a | 1 | 1 |
| discussions | Theme 3c | 1 | 1 |
| education | Theme 1, 2a, 3b, 3c | 4 | 4 |
| educational programs | Theme 2, 3c | 1 | 1 |
| effectiveness of technology | Theme 2a | 1 | 1 |
| emotional and intellectual accommodations | Theme 1c, 2b | 1 | 1 |
| emotional regulation | Theme 1c | 1 | 1 |
| emotional wellbeing | Theme 1c | 1 | 1 |
| encouraging communication | Theme 2b | 1 | 1 |
| engaging | Theme 2b | 1 | 1 |
| enthusiasm | Theme 2b | 1 | 1 |
| exercise | Theme 1a | 1 | 1 |
| feedback | Theme 2b | 2 | 2 |
| free choice | Theme 3c | 1 | 1 |
| fun | Theme 2 | 3 | 3 |
| genetics | Theme 2b | 1 | 1 |
| healthy habits | Theme 1a, 1b, 3c | 1 | 1 |
| healthy lifestyle | Theme 1 | 1 | 1 |
| higher-power authorities | Theme 2b | 1 | 1 |
| holistic | Theme 1 | 5 | 5 |
| homework involvement | Theme 3c | 1 | 1 |
| inclusivity | Theme 2b | 1 | 1 |
| inconsistent program involvement | Theme 2b | 1 | 1 |
| individualised programs | Theme 3 | 1 | 1 |
| informing parents | Theme 3c | 1 | 1 |
| insufficient training | Theme 3b | 1 | 1 |
| interactive platforms | Theme 2a | 1 | 1 |
| lack of awareness | Theme 3c | 1 | 1 |
| lack of cultural differences observed | Theme 2b, 3c | 1 | 1 |
| lack of resources | Theme 3a | 1 | 1 |
| lack of staff support | Theme 3a, 3b | 1 | 1 |
| limited awareness | Theme 3c | 1 | 1 |
| limited resource | Theme 3a | 1 | 1 |
| manpower and support | Theme 3a, 3b | 1 | 1 |
| meal planning | Theme 1b, 2b | 1 | 1 |
| memory | Theme 1a | 1 | 1 |
| modelling behaviour | Theme 3c | 1 | 1 |
| need for professional training | Theme 3b | 1 | 1 |
| need for resource and professional support | Theme 3a, 3b | 1 | 1 |
| newsletters | Theme 3c | 1 | 1 |
| no major challenge | Theme 2b, 3c | 1 | 1 |
| nutrition | Theme 1b | 2 | 2 |
| observation | Theme 2b | 9 | 9 |
| online platforms | Theme 2a | 1 | 1 |
| parent education | Theme 3c | 1 | 1 |
| parent focus | Theme 3c | 1 | 1 |
| parental engagement | Theme 3c | 3 | 3 |
| parental involvement | Theme 3c | 5 | 5 |
| participation | Theme 2b, 3b | 1 | 1 |
| performance checks | Theme 2b | 1 | 1 |
| performance evidence | Theme 2b, 3c | 1 | 1 |
| personalised approaches | Theme 2b | 1 | 1 |
| physical activity | Theme 1a | 2 | 2 |
| physical and mental interconnectedness | Theme 1a, 1c | 1 | 1 |
| physical exercise | Theme 1a | 2 | 2 |
| professional development | Theme 3b | 1 | 1 |
| reducing stress | Theme 1c | 1 | 1 |
| resources | Theme 3a | 1 | 1 |
| respecting personal beliefs | Theme 3c | 1 | 1 |
| safe space | Theme 2b | 1 | 1 |
| scenario-based teaching | Theme 2b | 1 | 1 |
| shared vision | Theme 3c | 1 | 1 |
| simple communication | Theme 2b | 1 | 1 |
| simplicity | Theme 2b | 1 | 1 |
| simplifying languages | Theme 2b | 1 | 1 |
| socialising | Theme 1 | 1 | 1 |
| socioeconomic differences | Theme 3c | 1 | 1 |
| special needs | Theme 2b | 1 | 1 |
| staff training | Theme 3b | 1 | 1 |
| structured programs | Theme 3b | 1 | 1 |
| student disinterest | Theme 2b | 1 | 1 |
| student feedback | Theme 2b | 1 | 1 |
| student involvement | Theme 2b | 4 | 4 |
| student surveys | Theme 2b | 1 | 1 |
| support | Theme 2a, 3 | 1 | 1 |
| support from parents | Theme 3c | 1 | 1 |
| tailored approaches | Theme 2b | 1 | 1 |
| tailored programs | Theme 2b | 2 | 2 |
| teamwork | Theme 2b | 1 | 1 |
| technology | Theme 2a | 3 | 3 |
| time | Theme 3a | 1 | 1 |
| time constraints | Theme 3a | 1 | 1 |
| time management | Theme 3a | 2 | 2 |
| tools for educators | Theme 2a | 1 | 1 |
| training | Theme 3b | 6 | 6 |
| training and resources | Theme 3a, 3b | 2 | 2 |
| training programs | Theme 3b | 1 | 1 |
| use of technology | Theme 2a | 1 | 1 |
| using technology and visual aids | Theme 2a | 1 | 1 |
| varying acceptance | Theme 3c | 1 | 1 |
| virtual reality | Theme 2a | 1 | 1 |
| voluntary participation | Theme 3c | 1 | 1 |
| workshops | Theme 3b, 3c | 3 | 3 |

Supplementary Material C. Thematic map of study’s themes and subthemes


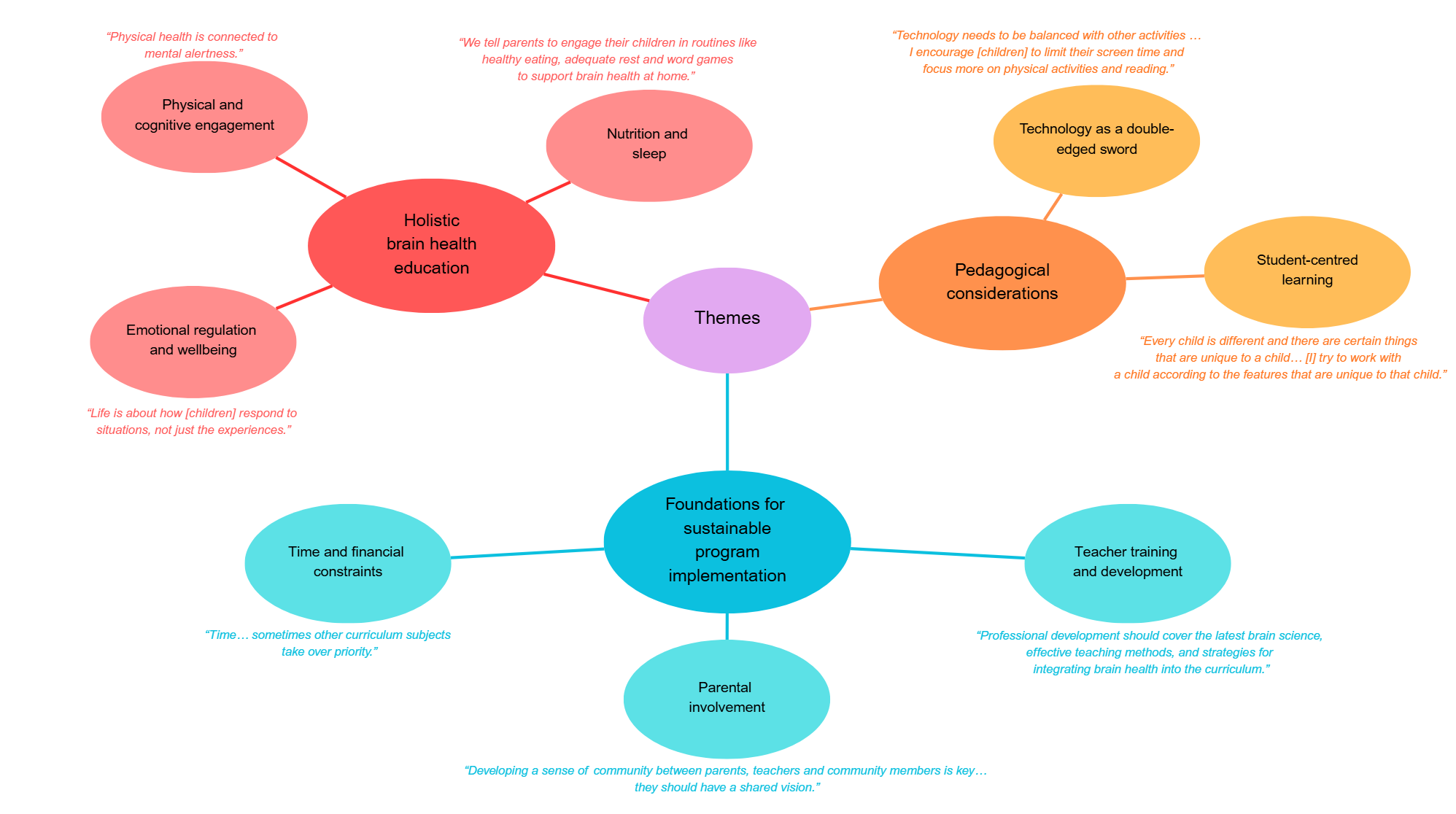


Supplementary Material D. Demographic information of participants across experience level, teaching stages, and school types (N = 25).

| **Variable** | **Age**  **(M [SD], Range)** | **Gender** | **Marital status** | **Number of children in family**  **(M [SD], Range)** | **Age of  children in family**  **(M [SD], Range)** | **Years  spent  teaching**  **(M [SD], Range)** | **Size  of  school**  **(M [SD], Range)** | **Number of students taught**  **(M [SD], Range)** |
| --- | --- | --- | --- | --- | --- | --- | --- | --- |
| Highest level of education |  |  |  |  |  |  |  |  |
| Bachelors (n = 18) | 28.83 (2.33), 24–35 | 13 females, 5 males | 6 single, 12 married | 1.06 (1.16),  0–4 | 3.03 (2.86), 0–9 | 5.44 (2.12), 2–10 | 777.78 (521.09), 300–2000 | 21.28 (8.72), 11–50 |
| Masters (n = 7) | 30.14 (7.60), 24–47 | 5 females, 2 males | 3 single, 1 de facto, 3 married | 0.71 (0.76), 0–2 | 4.00 (5.86), 0–16 | 6.14 (3.08), 2–10 | 535.71 (124.88), 350–700 | 23.64 (6.13), 14–30 |
| Grade/s currently teaching |  |  |  |  |  |  |  |  |
| Stage 1 (Years 1-2) (n = 11) | 28.73 (2.83), 24–35 | 11 females | 2 single, 9 married | 1.09 (1.22), 0–4 | 3.16 (3.13), 0–9 | 4.64 (1.86), 2–8 | 859.09 (633.96), 300–2000 | 18.05 (5.68), 11–30 |
| Stage 2 (Years 3-4) (n = 14) | 29.29 (5.51), 24–47 | 9 females, 5 males | 7 single, 1 de facto, 6 married | 0.79 (0.97), 0–3 | 3.06 (4.46), 0–16 | 6.36 (2.79), 2–10 | 664.29 (419.90), 300–2000 | 26.18 (8.13), 15–50 |
| Stage 3 (Years 5-6) (n = 11) | 30.25 (0.96), 29–31 | 2 females, 2 males | 1 single, 4 married | 1.00 (1.41), 0–3 | 2.58 (2.99), 0–5 | 7.00 (1.83), 5–9 | 675.00 (236.29), 500–1000 | 28.38 (14.74), 18–50 |
| All stages (K-6) (n = 1) | 31 | 1 female | Married | 0 | 0 | 8 | 500 | 25 |
| Type of school |  |  |  |  |  |  |  |  |
| Private (n = 8) | 30.63 (7.33), 24–47 | 6 females, 2 males | 3 single, 5 married | 1.13 (0.83), 0–2 | 4.69 (5.15), 0–16 | 5.50 (2.93), 2–10 | 743.75 (588.85), 300–2000 | 20.56 (6.97), 13–30 |
| Catholic (n = 2) | 27.00 | 1 females, 1 males | 1 single, 1 married | 0.50 (0.71), 0–1 | 2.00 (2.83), 0–4 | 7.50 (3.54), 5–10 | 475.00 (35.36), 450–500 | 21.94 (8.03), 11–50 |
| Public (n = 15) | 28.73 (1.75), 24–31 | 11 females, 4 males | 5 single, 1 de facto, 9 married | 0.96 (1.22), 0–4 | 2.74 (3.08), 0–9 | 5.47 (2.00), 2–9 | 723.33 (418.70), 350–2000 | 22.80 (9.05), 11–50 |

Supplementary Material E. Table of quotes for each subtheme.

| **Main themes** | **Subthemes** | **Quotes** |
| --- | --- | --- |
| 1. Holistic brain health education | 1. *Physical and cognitive engagement* | - “Physical activities and playing games that stimulate the brain are the most effective strategies for encouraging healthy behaviours.” - “Physical health is connected to mental alertness.” - “I see a positive response from students when they engage in physical activity before class.” |
|  | 1. *Nutrition and sleep* | - “By making students know the kind of food they should eat, the importance of exercise and having adequate sleep… the brain will be able to function.” - “My major struggle is with kids that have been struggling with unhealthy eating habits… they’ve been indulged for very long.” |
|  | 1. *Emotional regulation and wellbeing* | - “Life is about how [children] respond to situations, not just the experiences.” - “We teach the students about different parts of the brain… and strategies to manage emotions like breathing exercises.” - “Punching pillows, writing things down… it’s actually okay.” |
| 1. Pedagogical considerations | 1. *Technology as a double-edged sword* | - “Technology can be leveraged through digital learning tools, interactive simulations, and online resources that make brain health engaging and accessible.” - “Exciting pictures and diagrams are very attention-catching for kids.” - “With the use of AI, brain health can be enhanced, promoting social and emotional wellbeing.” - “Technology needs to be balanced with other activities … I encourage them to limit their screen time and focus more on physical activities and reading.” |
|  | 1. *Student-centred learning* | - “Every child is different and there are certain things that are unique to a child… what I basically do is try to work with a child according to the features that are unique to that child.” - “Yes, absolutely, [students] should be involved because they are the ones who are learning, and they have the right to know and understand.” - “I don’t involve students in decision-making because they might not have the best ideas for themselves. Teachers and parents know their strengths and weaknesses.” - “I ensure programs for students with disabilities are adapted through one-on-one interaction, employing trained personnel, and tailoring the curriculum to meet their needs.” |
| 1. Foundations for sustainable program implementation | 1. *Time and financial constraints* | - “We are very time poor. We’re under a lot of pressure. We’re constantly testing then giving feedback. Maths and English are priorities, and we have to fit so many extra things.” - “Time… sometimes other curriculum subjects take over priority.” - “Funding is very important… because you know, as a teacher, you can’t do that out of your pocket.” - “Cost is a big one at the moment because budgeting is really tight and that puts strain on the school and the principal to make cuts in areas that they think are not as important, whereas others might think that they are very important… not just the training of staff, but the lack of staff.” |
|  | 1. *Teacher training and development* | - “Like we did this rock and water training back in term three last year, and it’s never been mentioned again. So, when new staff come in, they wouldn’t have ever heard of it, so I think that’s definitely a challenge.” - “We need training, but we also need more staff to help run these programs.” - “Training of staff is important as well as having enough time and resources to implement programs effectively.” - “Professional development should cover the latest brain science, effective teaching methods, and strategies for integrating brain health into the curriculum.” - “Attending seminars on brain health, earning certifications in child psychology, and participating in online sources can help teachers stay informed.” |
|  | 1. *Parental involvement* | - “You engage the parent by giving homework that involves them… a parent could be obliged to support it.” - “We highlight these issues during parent-teacher association meetings, teaching the parents as well so they can guide their children.” - “Developing a sense of community between parents, teachers and community members is key… they should have a shared vision.” |
